# Vertical stratification of adult mosquitoes (Diptera: Culicidae) within a tropical rainforest in Sabah, Malaysia

**DOI:** 10.1186/s12936-016-1416-1

**Published:** 2016-07-19

**Authors:** Hayley L. Brant, Robert M. Ewers, Indra Vythilingam, Chris Drakeley, Suzan Benedick, John D. Mumford

**Affiliations:** Faculty of Natural Sciences, Centre for Environmental Policy, Imperial College London, Silwood Park Campus, Buckhurst Road, Ascot, SL5 7PY UK; Department of Life Sciences, Faculty of Natural Sciences, Imperial College London, Silwood Park Campus, Buckhurst Road, Ascot, Berkshire SL5 7PY UK; Department of Parasitology, Faculty of Medicine, University of Malaya, 50603 Kuala Lumpur, Malaysia; Department of Immunology and Infection, Faculty of Infectious and Tropical Diseases, London School of Hygiene and Tropical Medicine, London, WC1E 7HT UK; Faculty of Sustainable Agriculture, Universiti Malaysia Sabah, Locked Bag No. 3, 90509 Sandakan, Sabah Malaysia

**Keywords:** *Anopheles balabacensis*, Vector, *Plasmodium knowlesi*, Deforestation, Malaysia

## Abstract

**Background:**

Malaria cases caused by *Plasmodium knowlesi,* a simian parasite naturally found in long-tailed and pig-tailed macaques, are increasing rapidly in Sabah, Malaysia. One hypothesis is that this increase is associated with changes in land use. A study was carried out to identify the anopheline vectors present in different forest types and to observe the human landing behaviour of mosquitoes.

**Methods:**

Mosquito collections were carried out using human landing catches at ground and canopy levels in the Tawau Division of Sabah. Collections were conducted along an anthropogenic disturbance gradient (primary forest, lightly logged virgin jungle reserve and salvage logged forest) between 18:00 and 22:00 h.

**Results:**

*Anopheles balabacensis*, a vector of *P. knowlesi*, was the predominant species in all collection areas, accounting for 70 % of the total catch, with a peak landing time of 18:30–20:00 h. *Anopheles balabacensis* had a preference for landing on humans at ground level compared to the canopy (p < 0.0001). A greater abundance of mosquitoes were landing in the logged forest compared to the primary forest (p < 0.0001). There was no difference between mosquito abundance in the logged forest and lightly logged forest (p = 0.554). A higher evening temperature (p < 0.0001) and rainfall (p < 0.0001) significantly decreased mosquito abundance during collection nights.

**Conclusions:**

This study demonstrates the potential ability of *An. balabacensis* to transmit *P. knowlesi* between canopy-dwelling simian hosts and ground-dwelling humans, and that forest disturbance increases the abundance of this disease vector. These results, in combination with regional patterns of land use change, may partly explain the rapid rise in *P. knowlesi* cases in Sabah. This study provides essential data on anthropophily for the principal vector of *P. knowlesi* which is important for the planning of vector control strategies.

**Electronic supplementary material:**

The online version of this article (doi:10.1186/s12936-016-1416-1) contains supplementary material, which is available to authorized users.

## Background

Malaria still remains a public health problem throughout tropical and sub-tropical regions of the world, with an estimated 214 million new cases and 438,000 deaths in 2015 [1]. Four *Plasmodium* species are recognised as causing human malaria; *Plasmodium falciparum, Plasmodium malariae, Plasmodium ovale* and *Plasmodium vivax*, but recently a fifth species, *Plasmodium knowlesi*, has been recognized as causing symptomatic malaria in humans [2–4]. *Plasmodium knowlesi*, transmitted by the forest-dwelling *Anopheles* from the Leucosphyrus group, is an emerging cause for zoonotic human malaria in Southeast Asia [3, 5–7]. Malaysia has had a successful malaria control programme, aimed to eliminate malaria by 2020, with marked reductions in reported cases of *P. falciparum* and *P. vivax*, but there has been a recent increase in *P. knowlesi* cases [8–11]. *Plasmodium knowlesi* is now the most common cause of malaria in the Malaysian state of Sabah, accounting for 62 % of all malaria incidences in 2013 and presenting a threat to malaria elimination [8].

It has been proposed that land use change, including deforestation, forest fragmentation and agricultural practices, has increased the incidence of *P. knowlesi* by increasing the encroachment of humans into previously forested areas, allowing a higher interaction between vectors and human and macaque hosts [9, 12]. The increase in *P. knowlesi* cases may also be underestimated due to misdiagnosis during microscopic examination [13, 14]. Microscopy of stained blood smears allows differentiation between species, but frequent misdiagnosis occurs in areas containing *P. falciparum*, *P. vivax* and *P. knowlesi* [14].

Monkeys, particularly the long-tailed macaque (*Macaca fascicularis*) and the pig-tailed macaque (*Macaca nemestrina*) found in Southeast Asia, are the two main natural hosts of *P. knowlesi* [15]. A study in Sabah showed nearly all patients with *P. knowlesi* malaria had a recent history of forest or forest-edge exposure, and had seen a monkey in the preceding month [16]. Most members of the Leucosphyrus group, from the genus *Anopheles*, feed primarily on monkeys in the canopy and are capable of transmitting various *Plasmodium* species [17]. *Anopheles balabacensis* is the predominant vector of human malaria in Sabah [18, 19], and has also been incriminated as a *P. knowlesi* vector [20, 21].

Most primates are arboreal. Although some species of chimpanzees, baboons and macaques rest and feed at ground level during the day, primates almost always sleep in the canopy during the night [22]. As *Anopheles* species generally bite between 6 p.m. and 6 a.m., primate roosting sites are potentially a key location for disease transmission between primate hosts. It is hypothesized that vectors are biting humans at ground level, but if given the opportunity, will bite at canopy level. This study expects that key vector species should be present in the disturbed forest habitats where people come into contact with monkeys, but it is unknown which species should expect to be present in primary forest.

Since transmission is increasing in Sabah, it is important to identify the *P. knowlesi* vectors present and understand their biting behaviour within forest habitats. While other vector assessments are ongoing in the Interior, West Coast, Kudat and Sandakan Divisions in Sabah, the Tawau Division has not been studied. This study was conducted to determine the vertical distribution of mosquitoes and their biting preference in Sabah, Malaysia.

## Methods

### Study site

The study was conducted in the Tawau Division of Sabah, Malaysia. Three areas were selected along a forest disturbance gradient; primary lowland dipterocarp rainforest (PF), virgin jungle reserve (VJR) and twice-logged disturbed dipterocarp rainforest (LF) with forest disturbance quantified using on-the-ground forest plots [23]. Primary forest survey points were selected in the vicinity of Danum Valley Field Centre (4°58′N, 117°42′E), located within the Danum Valley Conservation Area. This area consists of 43,800 ha of protected dipterocarp rainforest [24]. Virgin jungle reserve (4°40′N, 117°32′E) and logged forest survey points (4°41′N, 117°34′E) were selected within the Benta Wawasan oil palm plantation. The 45,601 ha area is a mixture of twice-logged rainforest, virgin jungle reserve, acacia and oil palm. The VJR, of 2200 ha, has been logged around the edge, but never logged in the steep interior [25]. Survey points were selected 500–1000 m from the VJR edge in locations that had undergone light logging. Logged forest survey points were in selectively twice-logged forest, logged during 1970s, 1990s–2000s resulting in the cumulative removal of ~180 m^3^ ha^−1^ of timber [26], and currently being further disturbed by additional salvage logging activity in surrounding areas. Further details of the project area are given by Ewers et al. [25].

Three survey points, with a minimum separation distance of 500 m, were selected in each area. One tree was selected at each point based on its accessibility into the canopy, low density of epiphytes and height. Visual tree assessments were carried out to make sure every tree was safe to climb. The trees selected ranged from a height of 15 m in the logged forest to 30 m in the virgin jungle reserve and primary forest. Survey points were a subset of those designated as part of the central sampling design of the ‘Stability of Altered Forest Ecosystems (SAFE) Project’, a large-scale fragmentation experiment, which is investigating the long-term effects of forest fragmentation [25].

### Data collection

All data collection was carried out from April to July 2014, and mosquitoes were collected using human landing catches at ground and canopy height between 18:00 and 22:00 h. Four consecutive nights of collections were carried out in PF and VJR, and five nights in LF, using a rotation of collectors. Access was gained into the canopy using line insertion to high branches [27], followed by the double rope climbing technique taught by Canopy Access Limited [28]. Canopy samples were collected at a height of two-thirds the average canopy height at that location (10–20 m). Ground and canopy collections were conducted simultaneously. The average canopy height surrounding each selected tree was calculated using a laser rangefinder.

The collectors, with the aid of a red torch-light, aspirated mosquitoes off their own legs. Collected mosquitoes were placed into cups covered with a net cloth, and a new cup was used during every half an hour of collection. Mosquitoes were taken back to the field laboratory to be killed and sorted into individual tubes with silica gel. All mosquitoes were identified morphologically using keys [29–35].

### Meteorological data

Air temperature (°C) and relative humidity (%) were measured at each site using microclimate dataloggers at the base and in the canopy of each tree during sampling. Nightly rainfall (mm) data were obtained from the nearest rain gauges located at SAFE Project and Danum Valley Field Centre for the duration of the field survey (March to July 2014; range 1.0–3.5 km from sample sites). In addition, lunar illumination (%), cloud cover and unusual climatic events (e.g. strong winds) were recorded every half an hour by the collectors.

### Data analysis

Analyses were performed using R version 3.1.1 [36]. Simpson and Shannon diversity indices were calculated in each area using the vegan function ‘diversity’ (R package vegan, ‘diversity’) [37]. Species accumulation curves were calculated in each area using the vegan function ‘specaccum’ (R package vegan, ‘specaccum’) [37]. To estimate the number of undetected species and add them to the observed richness, true richness estimators were used. These estimators included the Chao species estimator (Chao 1) and Abundance Coverage Estimator (ACE). Chao 1 and ACE were used to estimate the extrapolated species richness in each area using ‘chao1’ and ‘ACE’ functions in the R package ‘fossil’ (R package fossil, ‘chao1’, ‘ACE’) [38].

The effect of canopy height (a dichotomous variable representing ground or canopy level) and forest disturbance (PF, VJR and LF) on mosquito abundance was analysed using a generalized linear mixed-effect model (R package lme4) [39], using day and site as random factors, with Poisson error distribution. Chi squared tests were used to compare the relative abundance of vector and non-vector species in each area, and between ground and canopy level. Differences in community composition at ground and canopy height were explored using Detrended Correspondence Analysis (DCA, vegan function ‘decorana’) [37]. The number of mosquitoes landing per night was used as a measure of relative abundance. Significant differences were tested for in community composition using a linear model with the first DCA axis as the response variable against canopy height and forest disturbance.

## Results

### Mosquito abundance

A total of 807 mosquitoes were collected from 39 human landing catch nights, consisting of 743 (92.1 %) anophelines, and 64 (7.9 %) culicines. A total of 555 (68.8 %) mosquitoes from 21 species were found at ground level in comparison to 252 (31.2 %) mosquitoes from 10 species at canopy level. *Anopheles balabacensis* was the predominant species at ground (62.4–87.5 % of all individuals) and canopy level (43.4–73.5 %) at each collection site. A full list of species is given in Table 1.

**Table 1:** Mosquitoes collected from different collection sites in the district of Tawau, Sabah, Malaysia

Mosquito genera and species	Number collected at:					
	Primary forest		Virgin jungle reserve		Logged forest	
	Ground	Canopy	Ground	Canopy	Ground	Canopy
*Am. orbitae*	0	0	0	0	1	0
*An.* Aitkenii group	0	0	21	0	18	0
*An. barbirostris*	0	0	1	0	3	0
*An. sp.* ^*a*^	0	0	1	0	10	3
*An. balabacensis*	21	12	83	23	296	130
*An. latens*	0	1	1	5	1	1
*An. macarthuri*	0	1	10	4	16	5
*An. maculatus*	1	0	7	2	12	4
*An. watsonii*	1	6	3	15	14	11
*Arm. confusus*	0	0	1	0	0	0
*Arm. jugraensis*	0	0	0	0	5	0
*Arm. sp.* ^*a*^	0	0	1	0	0	0
*Col. pseudotaeniatus*	0	0	1	0	0	0
*Coq. crassipes*	0	0	0	1	0	1
*Cx. sitiens*	0	0	0	0	5	1
*Cx. vishnui*	0	0	0	0	2	0
*Cx. (Lophoceraomyia)*	0	0	0	0	1	0
*Do. ganapathi*	1	2	0	3	4	16
*Pr. ostentatio*	0	0	1	0	0	0
*Ph. prominens*	0	0	0	0	1	0
*Stg. albopicta*	0	0	0	0	4	0
*Stg. sp.* ^*a*^	0	0	1	0	4	2
*Ve. sp.* ^*a*^	0	0	1	0	1	3
Total mosquitoes	24	22	133	53	398	177
No. of collection nights	12	12	12	12	15	15
Mosquitoes/nights	2	1.8	11.1	4.42	26.5	11.8

^a^Couldn’t be identified to species level

The number of species collected, Shannon index, Simpson index, Chao1 and ACE varied across forest disturbance and between ground and canopy level (Tables 2, 3). The Chao 1 and ACE predicted a higher number of species in the logged forest than the primary forest, and a higher number of species at ground level (Tables 2, 3). These patterns were also seen with the number of species collected, Shannon index and Simpson index. The species accumulation curves did not reach an asymptote at ground and canopy level, indicating not all species of mosquitoes had been collected (see Additional file 1).

**Table 2:** Mean species richness and diversity indices (±SE) of mosquito communities, collected at ground level

Forest disturbance	Human landing catches					
	N	Species no.	Shannon index	Simpson index	Chao1	ACE
PF	12	4	0.06 (0.04)	0.45 (0.14)	7	7
VJR	12	14	0.70 (0.10)	0.40 (0.05)	50	19.4
LF	15	18	0.88 (0.12)	0.43 (0.06)	30.5	19.8

Mean species richness and diversity indices (±SE) of mosquito communities, collected at ground level using human landing catches, in primary forest *PF*, virgin jungle reserve *VJR* and logged forest *LF*

**Table 3:** Mean species richness and diversity indices (±SE) of mosquito communities, collected at canopy level

Forest disturbance	Human landing catches					
	N	Species no.	Shannon index	Simpson index	Chao1	ACE
PF	12	5	0.24 (0.13)	0.65 (0.12)	7	6
VJR	12	7	0.71 (0.15)	0.50 (0.09)	7.5	7.36
LF	15	11	0.56 (0.11)	0.32 (0.06)	15.5	12.41

Mean species richness and diversity indices (±SE) of mosquito communities, collected at canopy level using human landing catches, in primary forest *PF*, virgin jungle reserve *VJR* and logged forest *LF*

### Effect of height and forest disturbance on mosquito abundance

**Fig. 1:**
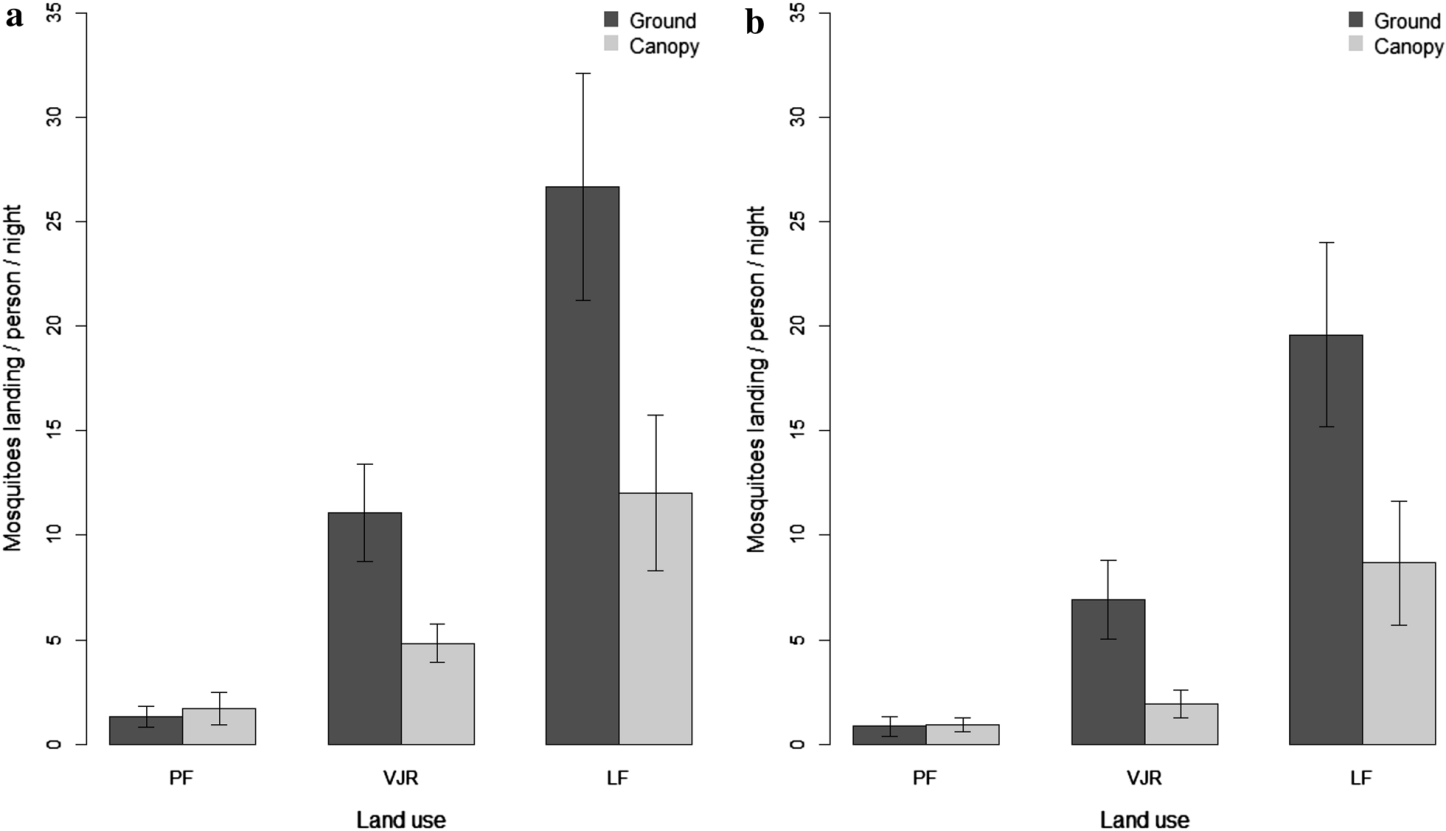
Effects of collection height on the human landing rate across a forest disturbance gradient. Effects of collection height on the human landing rate (number of mosquitoes per night per bait) across a forest disturbance gradient: primary forest (*PF*), lightly logged virgin jungle reserve (*VJR*), and twice-logged forest (*LF*). **a** Total abundance of all species combined, **b** Abundance of the most common species, *Anopheles balabacensis*, alone. *Error bars* show ± SE of the mean

**Fig. 2:**
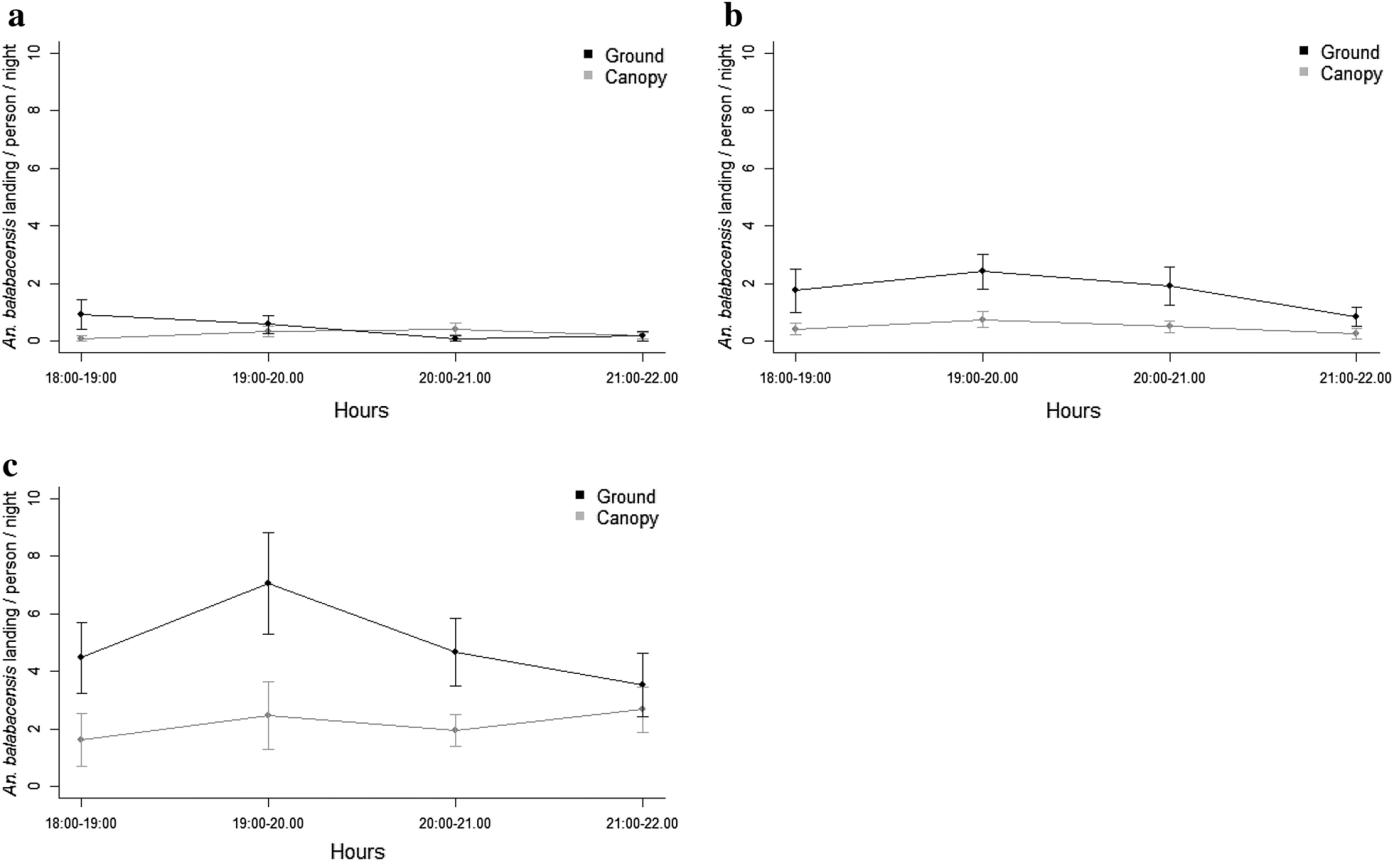
Hourly number of *Anopheles balabacensis* landing per person per night. Hourly number of *Anopheles balabacensis* landing per person per night, at ground and canopy level, across an anthropogenic disturbance gradient from **a** Primary forest, **b** Virgin jungle reserve and **c** Logged forest. *Error bars* show ± SE of the mean

Mosquito abundance in the canopy was significantly lower than at ground level (χ^2^ = 81.89, df = 1, p < 0.0001) (Table 4; Fig. 1a). Logged forest had a higher abundance than virgin jungle reserve or primary forest (χ^2^ = 10.94, df = 2, p < 0.05) (Fig. 1a). Similar patterns were seen with abundance of *An. balabacensis* (Table 4; Fig. 1b). Rainfall and a higher evening temperature decreased mosquito abundance (χ^2^ = 14.55, df = 1, p < 0.0001), whereas moonlight increased the abundance (χ^2^ = 4.49, df = 1, p < 0.05) (Table 4). Collector identity had no effect on mosquito abundance (χ^2^ = 3.871, df = 2, p = 0.144). Peak biting of *An. balabacensis* during the collection period was observed between 19:00 and 20:00 h in logged forest and virgin jungle reserve (Fig. 2).

**Table 4:** Effects of parameters on mosquito abundance

Predictor	All species				*Anopheles balabacensis*			
	β	SE	z	p	β	SE	z	p
Intercept	10.367	1.241	8.356	<0.0001***	10.233	1.423	7.190	<0.0001***
Height	−0.050	0.006	−8.804	<0.0001***	−0.060	0.007	−8.348	<0.0001***
Area PF	−2.268	0.545	−4.165	<0.0001***	−2.147	0.676	−3.176	0.001**
Area VJR	−0.307	0.518	−0.593	0.554	−0.633	0.657	−0.965	0.335
Temperature	−0.295	0.047	−6.330	<0.0001***	−0.307	0.053	−5.788	<0.0001***
Rainfall	−0.427	0.100	−4.306	<0.0001***	−0.448	0.115	−3.883	<0.0001***
Moonlight	0.006	0.003	2.129	0.033*	0.007	0.003	2.507	0.012*

Effects of height, area and habitat characteristics on daily mosquito abundance of all species combined, and on *Anopheles balabacensis* abundance separately in primary forest *PF*, virgin jungle reserve *VJR* and logged forest *LF*. Coefficient estimates (β), standard errors, associated Wald’s z-score, and p values are given

### Community composition

**Fig. 3:**
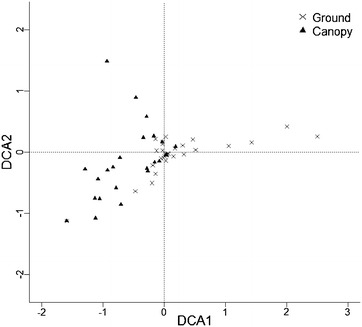
Detrended correspondence analysis for adult mosquito abundance at ground level and in the canopy. Detrended correspondence analysis (*DCA*) plot showing the major axes of variation for adult mosquito abundance at ground level and in the canopy of a tropical rainforest. The two axes represent linear summaries of the variation in the species numbers and areas

The DCA plot indicated that the community composition of the canopy was significantly different to ground level collection and across forest disturbance, with the community from the two height strata being strongly separated on the first axis (F_1, 60_ = 24.72, p < 0.0001, Fig. 3). The communities in the three categories of forest disturbance were not separated along the first axis (F_2, 60_ = 0.92, p = 0.4), but the interaction between height and forest disturbance was significant (F_2, 60_ = 8.37, p < 0.001). The first two axes accounted for 66.9 % of the total variance. *Anopheles balabacensis* was prevalent at both ground and canopy level, but the ground level community also included species such as *Anopheles barbirostris, Armigeres jugraensis*, *Culex vishnui* and the *Anopheles* Aitkenii group, which were not present in the canopy. The species turnover that causes the difference in community composition between ground and canopy was driven by species that have not been reported to cause disease transmission.

## Discussion

Studying the anthropophily of simian malaria vectors in the canopy of tropical forests is essential because the hosts are primarily arboreal. In order to develop, sustain or adapt a good control programme, it is important to monitor mosquito populations as well as their hosts and host-seeking preference, distribution and behaviour. Although previous studies in Southeast Asia have used monkey-baited traps at different canopy heights [12, 40, 41], this is the first study to attempt human landing catches, using this method, in the canopy. This study found that there was a higher abundance and human landing rate of mosquitoes at ground level, where people tend to be, than in the canopy where the simian hosts reside. This trend was driven by *An. balabacensis,* a key malaria vector in Sabah, and highlights the potential importance of this species in transmitting *Plasmodium* species from simian to human hosts.

*Anopheles balabacensis* was the most abundant mosquito in all sampled areas, accounting for 70 % of all collected species. *Anopheles balabacensis* is considered the most important vector of human malaria parasites on Banggi Island and mainland Sabah, Malaysia [18, 19, 30, 42]. In Sabah, *An. balabacensis* was found to be mainly exophagic, but could also be endophagic and exophilic [30, 43]. These behaviours cause *An. balabacensis* to be an effective vector of *P. knowlesi* from human to primate hosts. There were also two distinct subpopulations, one more zoophilic and one more anthropophilic [44–46]. *Anopheles balabacensis* occurs in forested areas, and readily bites human and monkey hosts, making it an ideal vector of simian malaria [20, 47].

Currently insecticide-treated bed nets and indoor residual spraying are the two main control methods in Malaysia [48]. This study showed *An. balabacensis* bites as early as 18:00 h in the Tawau Division. Other studies have shown the species bites as early as 18:00 h in recent years in comparison to late night biters in previous decades [44, 49–51]. Given that *An. balabacensis* is early evening biting, highly anthropophilic, exophagic and exophilic, current control methods are not sufficient to break the transmission cycle of *P. knowlesi* [52]. In Vietnam and Cambodia, long-lasting insecticidal hammocks (LLIH) were shown to reduce malaria incidence and prevalence in forested areas, and may prove to be an additional effective tool in reduction of malaria in Malaysia [52–54]. The use of repellents have been used for malaria control, but need to be tested in forest and plantations areas.

This study found a different community composition of mosquitoes in the canopy to that at ground level. Different mosquito species have particular flight distributions, with certain species flying and feeding close to ground, some species showing a preference for higher canopy heights, while others show a random distribution [55, 56]. The percentage biting at different canopy heights can be affected by microclimate conditions, such as relative humidity, temperature, wind speed and rainfall [57, 58], but may also change according to time of day [59].

Moonlight appeared to have a significant impact on mosquito activity, with human landing rates increasing on bright nights. Although some studies have shown moonlight increases relative abundance of biting vectors [60–64], others have shown a decrease [65–68] or no effect at all [69]. Collection bias was reduced in this study by collecting in each area under different phases of the moon.

This study also showed how forest disturbance affected mosquito abundance, species richness and human landing rates. Vector abundance was greater in the lightly modified virgin jungle reserve and heavily modified logged forest than in the unmodified primary forest. These results may be explained by the availability of larval breeding sites. Wheel tracks in logged areas due to logging activities can provide breeding sites for a range of mosquito species, whereas wheel tracks are not present within primary forests or virgin jungle reserves [31]. Species richness, estimated by the Chao1 index and ACE, differed across forest disturbance and height, with logged forest and ground level having a higher species richness than primary forest and canopy.

## Conclusions

This study has given an overview of mosquito species found in the Tawau Division, including the anthropophily of *P. knowlesi* vectors at canopy and ground levels. *Anophelesbalabacensis* was the predominant species found in primary forest, virgin jungle reserve and logged forest with a preference for landing on humans at ground level. As *An. balabacensis* is a vector of human and simian malaria, these findings will be useful for the planning of control strategies of malaria vectors.

## Additional file

10.1186/s12936-016-1416-1 Species accumulation curves for human landing catch sampling in primary forest at (a) Ground and (b) Canopy level, virgin jungle reserve at (c) Ground and (d) Canopy level, and logged forest at (e) Ground and (f) Canopy level. Shaded area indicates 95 % confidence intervals.

## Abbreviations

ACE: abundance coverage estimator
DCA: detrended correspondence analysis
LF: logged forest
LLIH: long-lasting insecticidal hammocks
PF: primary forest
VJR: virgin jungle reserve
