# Supplementary figures and images for: Vertical stratification of adult mosquitoes (Diptera: Culicidae) within a tropical rainforest in Sabah, Malaysia
Source: Malar J. 2016 Jul 19;15:370. doi: 10.1186/s12936-016-1416-1 (PMC4950076; doi:10.1186/s12936-016-1416-1)

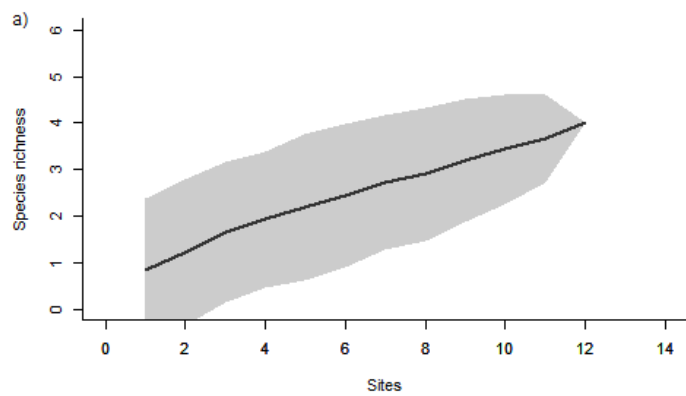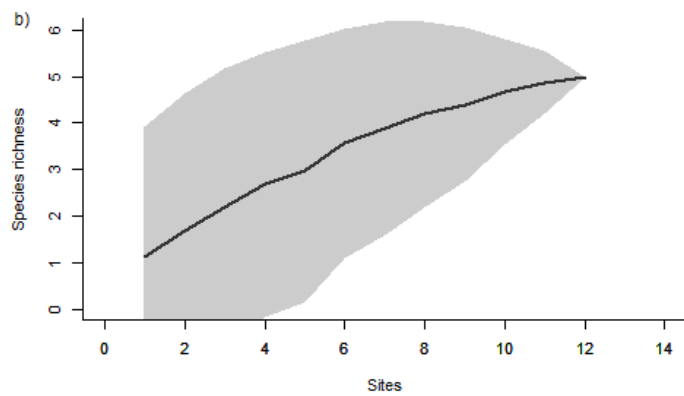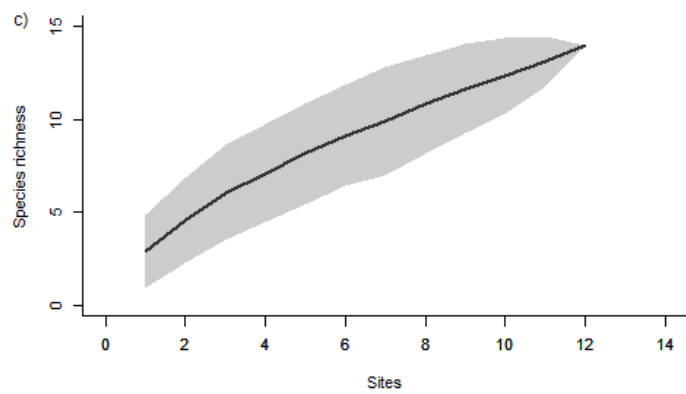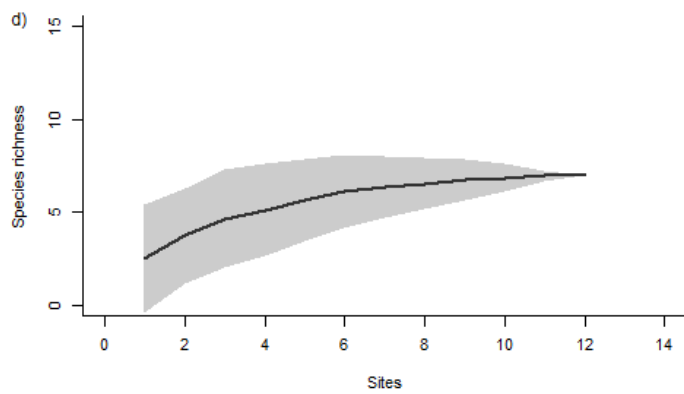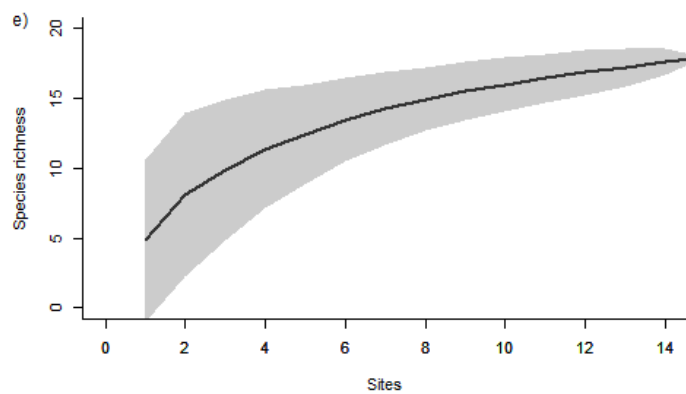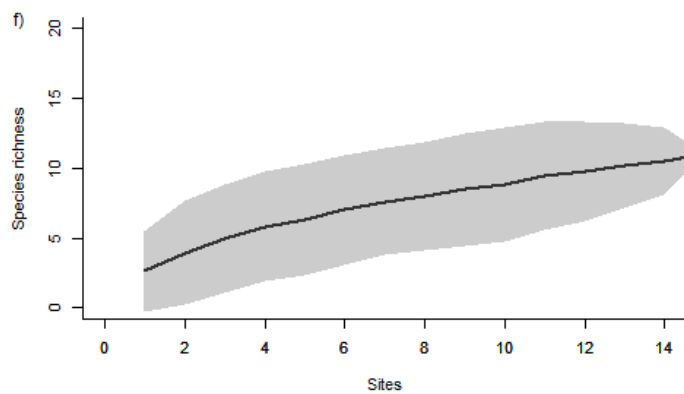

Supplement: Supplementary file 1 — 10.1186/s12936-016-1416-1 Species accumulation curves for human landing catch sampling in primary forest at (a) Ground and (b) Canopy level, virgin jungle reserve at (c) Ground and (d) Canopy level, and logged forest at (e) Ground and (f) Canopy level. Shaded area indicates 95 % confidence intervals. [file 12936_2016_1416_MOESM1_ESM.pdf]
